# Mutual Balance between Vasohibin-1 and Soluble VEGFR-1 in Endothelial Cells

**DOI:** 10.3390/ph4060782

**Published:** 2011-05-31

**Authors:** Hiroki Miyashita, Hirotada Suzuki, Akihide Ohkuchi, Yasufumi Sato

**Affiliations:** 1 Department of Vascular Biology, Institute of Development Aging and Cancer, Tohoku University, 4-1 Seiryo-machi, Aoba-ku, Sendai 980-8575, Japan; 2 Department of Obstetrics and Gynecology, Jichi Medical University School of Medicine, Japan

**Keywords:** angiogenesis inhibitor, endothelial cell, VASH1, sVEGFR-1

## Abstract

Vasohibin-1 (VASH1) is a VEGF-inducible gene of endothelial cells (ECs) that acts as a negative feedback regulator of angiogenesis. To further characterize the function of VASH1, we transfected human VASH1 gene into the mouse EC line MS1, established stable VASH1 expressing clones, and determined gene alteration by cDNA microarray analysis. Among the various angiogenesis-related genes, vascular endothelial growth factor type 1 receptor (VEGFR-1) and its alternative spliced form, soluble VEGFR1 (sVEGFR-1), were found to be the most significantly down-regulated genes. Transient overexpression of VASH1 in human umbilical vein endothelial cells confirmed the down-regulation of VEGFR-1 and sVEGFR-1. sVEGFR-1 is a decoy receptor for VEGF and inhibits angiogenesis. Interestingly, when sVEGFR-1 was overexpressed in ECs, it inhibited the expression of VASH1 in turn. These results suggest that VASH1 and sVEGFR-1, two angiogenesis inhibitors, mutually balance their expressions in ECs.

## 1. Introduction

Angiogenesis, the formation of neovessels, is involved in both physiological and pathological conditions. Angiogenesis is regulated by a local balance between stimulators and inhibitors. Angiogenesis stimulators include vascular endothelial growth factor (VEGF), whereas angiogenesis inhibitors include thrombospondins, pigment epithelium derived factor, angiostatin, endostatin, and so forth [1].

The VEGF family consists of five members; VEGF-A, VEGF-B, VEGF-C, VEGF-D, and placenta growth factor (PlGF). There are also three VEGF receptor (VEGFR) tyrosine kinases; VEGFR-1, VEGFR-2 and VEGFR-3. Members of the VEGF family show different affinities for the receptors. VEGFR-1 is able to bind VEGF-A, VEGF-B and PlGF. VEGFR-2 is activated primarily by VEGF-A, but cleaved forms of VEGF-C and VEGF-D can also activate VEGFR-2. VEGFR-3 is activated by VEGF-C and VEGF-D. Vascular endothelial cells (ECs) express mainly VEGFR-1 and VEGFR-2, whereas lymphatic ECs mainly express VEGFR-3 in adults. VEGFR-2 is the major mediator of VEGF-A driven responses for angiogenesis. The binding-affinity of VEGFR-1 for VEGF-A is one order of magnitude higher than that of VEGFR2, whereas the kinase activity of VEGFR-1 is about 10-fold weaker than that of VEGFR-2 [2]. *VEGFR-1 (-/-)* mice died in utero because of overgrowth of ECs, but mice lacking the tyrosine kinase domain of VEGFR-1 remain healthy and have a normal vasculature [3,4]. Thus, the ligand binding domain of VEGFR-1 are sufficient for normal vascular development in embryo, most likely by sequestering VEGF-A from VEGFR-2.

We recently isolated vasohibin-1 (VASH1) from VEGF-A inducible genes in ECs that inhibits migration and proliferation of ECs in culture, and exhibits anti-angiogenic activity *in vivo* [5]. The expression of VASH1 in ECs is induced not only by VEGF-A but also by fibroblast growth factor 2 (FGF-2), another potent angiogenic factor [5,6]. Thus, VASH1 is thought to be a negative-feedback regulator of angiogenesis. Immunohistochemical analysis revealed that VASH1 protein is expressed selectively in ECs in the developing human or mouse embryo, is reduced in expression in the post-neonate, but is induced in ECs at the site of angiogenesis [7]. Analysis of the spatiotemporal expression and function of VASH1 during angiogenesis revealed that VASH1 is expressed not in ECs at the sprouting front but in ECs of newly formed blood vessels behind the sprouting front where angiogenesis is terminated [8]. The expression of VASH1 is evident in various pathological processes such as cancers [9-13], atherosclerosis [14], age-dependent macular degeneration (AMD) [15], diabetic retinopathy [16], and so forth. Moreover, when applied exogenously, VASH1 shows anti-angiogenic activity under various pathological conditions such as in tumors, arterial intimal thickening and retinal neovascularization [9,14,17]. However, the molecular mechanisms underlying angiogenesis inhibition by VASH1 remain to be characterized. Here we intended to characterize the target genes of VASH1 in ECs. Using cDNA microarray analysis of stable VASH1 expressing EC clones, we identified both full-length and soluble forms of VEGFR-1 as the target genes of VASH1 in ECs.

## 2. Materials and Methods

### 2.1. Cells

MS1, an immortalized cell line with a SV40 large T antigen from mouse pancreatic ECs [18], was purchased from American Type Culture Collection (Manassas, VA, USA). The cells were cultured in αMEM (Invitrogen, Carlsbad, CA, USA) supplemented with 10% fetal bovine serum (FBS, JRHBiosciences, San Antonio, TX, USA). Human umbilical vein endothelial cells (HUVECs) were obtained from KURABO (Osaka, Japan) and were cultured on type I collagen-coated dishes (IWAKI, Tokyo, Japan) containing endothelial basal medium-2 (EBM-2; Clonetics Corp., San Diego, CA, USA) supplemented with EC growth supplements and 2% FBS.

### 2.2. Establishment of VASH1 Expressing MS1 Clones

To improve the activity of transcription, we placed the CMV promoter of the pcDNA3.1/Hygro plasmid (Invitrogen) with the chicken β-actin promoter derived from pCALL2 [19]. This vector, pCALL2-pcDNA3.1/Hygro, was used for the transfection in this study. For the production of the VASH1 expression vector, the human VASH1 gene (5481 bp) containing the complete open reading frame (386 n.t.-1483 n.t.) [5] was cloned into the pCALL2-pcDNA3.1/Hygro vector at multiple cloning sites (Xho-I and Not-I). MS1 cells were transfected with the VASH1 expression vector by using Effectene transfection reagent (Qiagen, Valencia, CA, USA) according to the manufacturer's protocol. After the transfection, the cells were selected by hygromycin (500 μg/mL, Invitrogen). Following the selection, the cells were seeded at 0.3 cells per well in 96 well plates with 100 μL of culture medium in each well. The cells were later expanded into larger wells.

### 2.3. Gene Transfer in HUVECs

A replication-defective adenovirus vector encoding the human VASH1 (AdVASH1) or the β-gal gene (AdLacZ) was prepared as described previously [5]. The replication-defective adenovirus vector encoding the human VEGFR-1 gene (AdVEGFR-1) was a generous gift from Masabumi Shibuya (Tokyo Medical and Dental University). The HUVECs were infected with the adenovirus vectors at a multiplicity of infection (MOI) of 10 to 100. After the infection, RNAs and proteins were extracted at 24 and 36 hours, respectively.

### 2.4. Reverse Transcriptase-Polymerase Chain Reaction (RT-PCR)

Total RNA was extracted by the acid guanidinium-phenol-chloroform method using ISOGEN (Nippon Gene, Toyama, Japan). RT-PCR was performed by using a one step RT-PCR kit (Invitrogen) according to the manufacturer's instructions. The following primer pairs were synthesized and used for amplification: the respective sense (S) and the antisense (AS) primer pairs used were as follows: mouse and human VASH1, 5'- ATGGACCTGGCCAAGGAAAT-3′ and 5'-CATCCTTCTTCCGGTC CTTG-3′; mouse VEGFR-1, 5'-GCGCATGACGGTCATAGAAG-3′ and 5'-CAGGTGTGGCGCTT CCGAAT-3′; human VEGFR-1, 5'-ATGGTCAGCTACTGGGACAC-3′ and 5'-GAATGACGAGCTC CCTTCCTT-3′; mouse sVEGFR-1, 5'-ACTCTCAGACCCCTGGAATC-3′ and 5'-GATCCGAGAGA AAATGGCCT-3′; human sVEGFR-1, 5'-CATCACTCAGCGCATGGCAA-3′ and 5'-CAGCCTTTTT GTTGCGTGC-3′; mouse and human G3PDH, 5′-ACCACAGTCCATGCCATCAC-3′ and 5′-TCCAC CACCCTGTTGCTGTA-3′. The PCR consisted of 27 cycles of 94 °C for 15 s, at 58 °C for 30 s, and finally at 68 °C for 30 s. The PCR products were electrophoresed through a 2% agarose gel containing 0.5 mg/mL ethidium bromide and visualized.

### 2.5. Western Blot Analysis

Western blot analysis was performed as described previously [20]. Briefly, extracted protein was separated by SDS-PAGE on a 7.5-10% separating gel and transferred to a Hybond-ECL membrane (Amersham, Buckinghamshire, UK). The membrane was incubated with anti-human VASH1 monoclonal antibody (Ab) [5], anti-VEGFR-1 Ab (Santa Cruz Biotechnology, Inc., CA, USA), anti-sVEGFR-1 Ab (Zymed Lab., San Francisco, CA, USA), or anti-ß-actin Ab (Sigma, St. Louis, MO, USA) as the primary antibody according to the manufacturer's instructions. The signal was visualized by using horseradish peroxidase-conjugated secondary antibodies and enhanced chemiluminescence (Immobilon Western, Millipore, Billerica, MA, USA) with a LAS-1000 image analyzer (Fuji Film, Tokyo, Japan).

### 2.6. Cell Proliferation

Cells were inoculated at a density of 1 × 10^4^ per well into 100 mm dishes, and cultured in 10% FCS/αMEM. After incubation for the desired period, the cells were counted with a hemocytometer.

### 2.7. Cell Migration

Cells were harvested with trypsin-EDTA, resuspended in 10% FCS/αMEM in a final volume of 100 μL, loaded (5 × 10^4^ cells per well) into the upper chamber of a Transwell Polycarbonate Membrane (pore size: 8 μm; Costar, Cambridge, MA, USA) containing 600 μL of 10% FCS/αMEM in the lower chamber, and incubated at 37 °C for 4 hours. Cells on the lower surface were stained with the reagents from a Diff Quick kit (International Reagents, Kobe, Japan) and counted.

### 2.8. cDNA Microarray Analysis

Total RNA was isolated from the mock control and clone 4 by ISOGEN according to the manufacturer's instructions. The RNAs were reverse-transcribed in the presence of Cy3 or Cy5-labeled CTP, respectively. The labeled probes were hybridized to a Filgen Array Mouse 32K (Oxford Gene Technology, Oxford, UK) containing 31,769 genes, and the signals were detected by use of a GenePix 4000B (Olympus, Tokyo, Japan). Genes with a Cy5 signal/Cy3 signal ratio <2.0 or > 0.5 were considered to have changed in activity.

### 2.9. Blood Pressure and Urinary Albumin Excretion of Mice

Animal studies were reviewed and approved by the committee for animal study at our institute in accord with established standards of humane handling. AdVASH1, AdVEGFR-1 or AdLacZ (1 × 10^9^ plaque-forming units [pfu]) was intravenously injected into a tail vein of BALB/c mice (Charles River Laboratories Japan, INC.). Before and seven days after the viral injection, mean blood pressure of conscious mice was measured by the tail cuff method (BP-98A; Softron Co. Ltd., Tokyo, Japan) according to the manufacture's instruction. Seven days after the viral injection, mice were put in mouse metabolic cages (Metabolica type MM; Sugiyama-Gen Iriki Co. Ltd., Tokyo, Japan) and urine was collected for successive eight hour periods. Urine was centrifuged at 2,000× *g* and the urinary albumin level was determined using an ELISA kit (Albuwell M; Exocell, Philadelphia, PA). Urinary creatinine levels were also measured by an ELISA kit (The Creatinine Companion; Exocell).

### 2.10. Calculations and Statistical Analysis

The statistical significance of differences in the data was evaluated by use of unpaired analysis of variance, and P values were calculated by the unpaired Student t test. P > 0.05 was accepted as statistically significant.

## 3. Results

**Figure 1:**
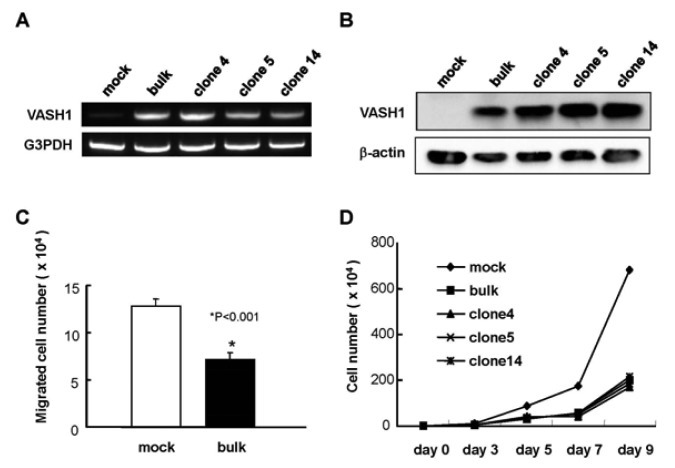
Establishment of VASH1 overexpressing MS1 clones.

We introduced the human VASH1 gene into MS1 cells, established bulk transfectants, and thereafter isolated 3 clones. First the expression of VASH1 mRNA and protein was determined (Figure 1A and B). We then examined the properties of the VASH1 expressing mouse endothelial clones. As expected, migration and proliferation were significantly decreased in these VASH1 transfectants (Figures 1C and D).

**Figure 2:**
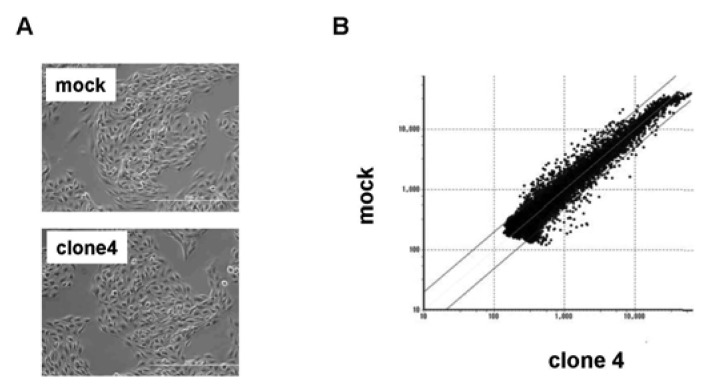
Scatter plot of mock vs. VASH1 over expression clone 4.

We used clone 4 (Figure 2A) for the following analysis, as it showed the highest expression of VASH1 (Figure 1A). Total RNA was isolated and the gene expression profile was compared with the mock control by cDNA microarray analysis (Figure 2B). The scatter plot of the mock control versus clone 4 is shown in Figure 2B. Among 31,769 mouse genes, 170 genes were increased by more than 200%, whereas 178 genes were decreased by less than 50% in clone 4. The top 20 up-regulated and down-regulated genes are listed in Table 1 and Table 2, respectively. We further evaluated the known angiogenesis-related genes. It was revealed that the expression of 7 genes (CCL2, CCL5, TIMP-1, COX-2, ARNT, CXCL1, and Jagged1) was augmented by more than 200%, whereas that of 2 genes (VEGFR-1 and Ets-1) was down-regulated by less than 50% (Table 3).

**Figure 3:**
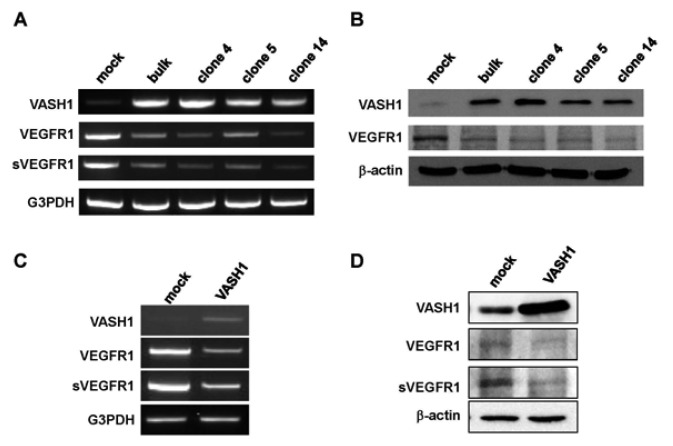
VASH1 inhibits VEGFR-1 and sVEGFR-1 expression in ECs.

Here we focused our attention on VEGFR-1, the most down-regulated gene. The characteristic feature of the VEGFR-1 gene is that it encodes not only a full-length membrane receptor but also a soluble form (sVEGFR-1) carrying the VEGF-binding domain as well as a 31-amino-acid stretch derived from an intron [22]. We therefore analyzed the expression of sVEGFR-1 as well. RT-PCR and Western blot analyses showed decrease expression of VEGFR-1 and sVEGFR-1 in all VASH1 expressing clones (Figures 3A and B). To further confirm these results, we transiently overexpressed VASH1 in HUVECs. RT-PCR and Western blot analyses also showed this decrease in the levels of VEGFR-1 and sVEGFR-1 (Figures 3C and D).

**Figure 4:**
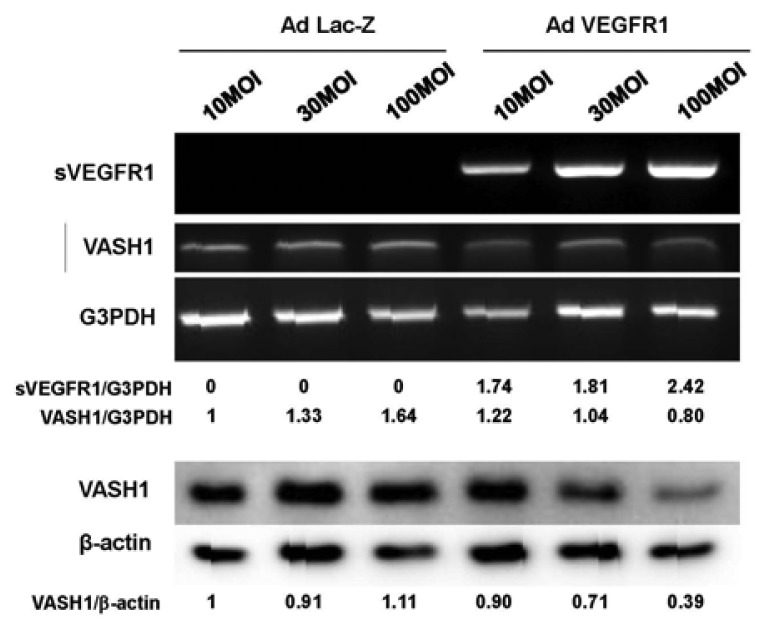
sVEGFR-1 inhibits VASH1 expression in ECs.

sVEGFR-1 is a decoy receptor and inhibits VEGF mediated signals. VASH1 is a VEGF-inducible angiogenesis inhibitor expressed in ECs. We therefore examined whether sVEGFR-1 affects the expression of VASH1 in ECs. To do so, we transiently overexpressed the sVEGFR-1 gene in HUVECs. RT-PCR and Western blot analyses demonstrated that sVEGFR-1 down-regulated the expression of VASH1 in HUVECs (Figure 4).

**Figure 5:**
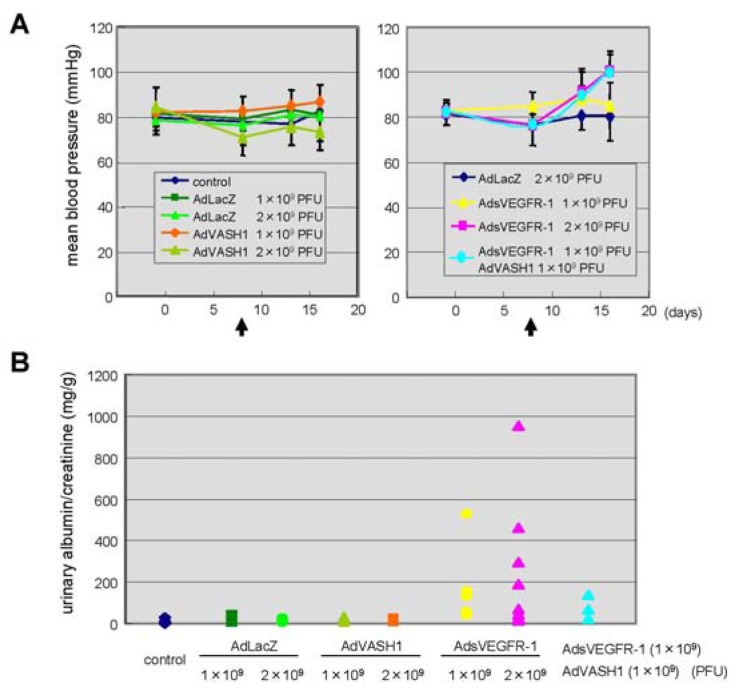
Different effects of sVEGFR-1 and VASH1 on blood pressure and urinary albumin excretion.

The blockade of VEGF mediated signals causes regression of normal quiescent vessels, hypertension and proteinuria [21]. Here we examined whether VASH1 caused hypertension and proteinuria. AdsVEGFR-1 increased mean blood pressure, but AdVASH1 did not (Figure 5A). Similarly AdsVEGFR-1 increased the urinary albumin excretion, but AdVASH1 did not (Figure 5B). AdVASH1 exhibited little effect on the increased mean blood pressure or urinary albumin excretion induced by AdsVEGFR-1 (Figure 5A and B).

## 4. Discussion

Here we analyzed the target genes of VASH1 in ECs, and revealed for the first time that VASH1 down-regulated the expression of both full-length and soluble form of VEGFR-1 in ECs. Interestingly, sVEGFR-1, a decoy receptor that blocks VEGF mediated signals, down-regulated the expression of VASH1 in return. Endogenous sVEGFR-1 is thought to inhibit angiogenesis by reducing VEGF-mediated angiogenic signals [22]. Thus, our present study indicates that these two factors mutually regulate their expression in ECs. We propose that VASH1 and sVEGFR-1 interact with each other within ECs for the fine tuning of angiogenesis.

The expression of VASH1 in ECs is known to be induced by VEGF-VEGFR2 and its downstream PKC-δ mediated signaling pathway [6]. We therefore think it reasonable that sVEGFR-1 would inhibit the expression of VASH1 in ECs. In contrast, the regulation of the expression of full-length and soluble form of VEGFR-1 is not well characterized. Further study is required to elucidate the mechanism as to how VASH1 down-regulates the expression of VEGFR-1 in ECs.

From the clinical experience of anti-angiogenic cancer treatment, it is now well recognized that the *in vivo* blockade of VEGF-mediated signals causes vascular complications including hypertension and proteinuria [21]. Indeed, the tail vein injection of AdsVEGFR-1 increased blood pressure and induced proteinuria (Figure 5). In contrast to the blockade of VEGF-mediated signals, we recently reported that VASH1 did not cause any vascular regression [23]. Here we extended our analysis on the vascular complication, and demonstrated that VASH1 did not cause hypertension or proteinuria. VASH1 could not prevent the hypertension or proteinuria induced by sVEGFR-1. Nevertheless, this finding that VASH1 caused neither hypertension nor proteinuria can be a merit of VASH1 when this inhibitor is applied as anti-angiogenic treatment.

In relation to vascular phenotypes of the blockade of VEGF-mediated signals, much attention is now being paid to preeclampsia. Preeclampsia is a disorder of gestation characterized by hypertension and renal dysfunction, and it is a major cause of maternal, fetal and neonatal mortality. Although the etiology of preeclampsia is still unclear, its major phenotypes, i.e., hypertension and proteinuria, may be due to an excess of circulating anti-angiogenic factors, most notably sVEGFR-1 [24]. We have previously shown that VASH1 is expressed in the vasculature of human placenta [6]. In this context, it would be interesting to examine the significance of VASH1 in normal pregnancy and patients with preeclampsia. Such study is currently under way.

## Figures and Tables

Establishment of VASH1 overexpressing MS1 bulk and clones was described in Materials and Methods. Total RNAs and proteins were extracted from each clone. RT-PCR (A) and Western (B) blot analysis for VASH1 was performed. (C) Migration was compared between mock and VASH1 transfectants. Means and SDs are shown. (D) Proliferation was compared between mock and VASH1 transfectants.

**(A)** Morphology of mock and VASH1 expressing clone 4 is shown. **(B)** RNAs were extracted from the mock and clone 4, and cDNA microarray analysis was performed as described in Materials and Method. Two lines indicate a 1:2 (lower) or 2:1 (upper) ratio between mock vs. VASH1 overexpressing clone 4.

Total RNAs and proteins were extracted from mock cells and from each VASH1 overexpressing clone, and RT-PCR (A) and Western blot analysis (B) for VEGFR-1 and sVEGFR-1 were performed as described in Materials and Method. The HUVECs were infected with the Lac-Z or VASH1 adenovirus vector at a multiplicity of infection (MOI) of 30. After the infection, total RNAs and proteins were extracted from vasohibin-1 over expression HUVECs. RT-PCR (C) and Western (D) blot analysis for VEGFR-1 and sVEGFR-1 was performed.

The HUVECs were infected with the AdsVEGFR-1 at the indicated a multiplicity of infection (MOIs). After the infection, total RNAs and proteins were extracted from sVEGFR-1 overexpressing HUVECs. RT-PCR (upper panel) and Western blot analysis (lower panel) for VASH1 were performed. Intensity of bands was quantified, and standardized values are shown.

**A**: Indicated PFU of adenovirus vectors were injected *via* a tail vain on day 8. Arrows indicate the timing of adenovirus injection. Mean blood pressure was assessed on day 0, *7*, 12, 16; means and SDs are shown. **B**: Indicated PFU of adenovirus vectors were injected. Twenty-four-hour urinary albumin excretion was quantified on day 7 after the adenovirus injection.

**Table 1:** Top 20 up-regulated genes in the VASH1 stable transfectant.

**Fold induction**	**Gene name**	**Accession No.**
8.07	Estradiol 17 beta-dehydrogenase 5	NM_030611
7.65	T-cell specific GTPase	NM_011579
6.16	Dematin	NM_013514
5.43	interferon-induced protein with tetratricopeptide repeats 3	NM_010501
4.91	Osteoactivin	NM_053110
4.63	BTB/POZ domain containing protein 9	NM_172618
4.29	interferon, alpha-inducible protein	NM_172618
4.26	Galectin-3	NM_010705
4.19	ADP-ribosylation factor-like 2 binding protein	NM_024191
3.79	potassium channel interacting protein 4	NM_030265
3.79	CCL2	NM_011333
3.78	olfactory receptor 576	NM_001001805
3.59	KELCH-like protein 4	NM_172781
3.41	interferon-induced protein 44	NM_133871
3.39	interferon, alpha-inducible protein 27	NM_029803
3.12	acyl-Coenzyme A thioesterase 2	NM_019736
3.28	stefin A2 like 1	NM_173869
3.11	guanylate nucleotide binding protein 3	NM_018734
3.09	2′-5′-oligoadenylate synthetase 1A	NM_145211
3.00	Male-specific lethal 3-like 1	NM_010832

**Table 2:** Top 20 down-regulated genes in the VASH1 stable transfectant.

**Fold induction**	**Gene name**	**Accession No.**
0.22	Phospholipid transfer protein	NM_011125
0.22	VEGFR1	NM_010228
0.28	Oligodendrocyte transcription factor 1	NM_016968
0.28	olfactory receptor 635	NM_147118
0.28	PHD finger protein 19	NM_028716
0.28	Trace amine receptor 1	NM_053205
0.29	Gastrokine 1	NM_025466
0.29	Mast cell carboxypeptidase A	NM_007753
0.30	poliovirus receptor-related 2	NM_008990
0.30	Versican core protein	XM_488510
0.30	Janus kinase 3	NM_010589
0.30	thrombopoietin	NM_009379
0.31	nudix	NM_025539
0.31	Anaplastic lymphoma kinase	NM_007439
0.33	Lymphocyte antigen 86	NM_010745
0.33	Fc receptor-like 3	NM_144559
0.34	Serine/threonine-protein kinase ULK1	NM_009469
0.34	Runt-related transcription factor 3	NM_019732
0.34	RAB GTPase activating protein 1	AK_044346
0.35	kidney expressed gene 1	NM_029550

**Table 3:** Altered expression of angiogenesis-related genes in the VASH1 transfectant.

**Gene name**	**Fold induction**
VEGFR1	0.22
Ets-1	0.45
Jagged1	2.06
CXCL1	2.17
ARNT	2.18
COX-2	2.38
TIMP-1	2.39
CCL5	2.45
CCL2	3.79
